# Activation of cortical 5-HT_3_ receptor-expressing interneurons induces NO mediated vasodilatations and NPY mediated vasoconstrictions

**DOI:** 10.3389/fncir.2012.00050

**Published:** 2012-08-10

**Authors:** Quentin Perrenoud, Jean Rossier, Isabelle Férézou, Hélène Geoffroy, Thierry Gallopin, Tania Vitalis, Armelle Rancillac

**Affiliations:** Laboratoire de Neurobiologie, CNRS UMR 7637, ESPCI ParisTechParis, France

**Keywords:** neurovascular coupling, mCPBG, serotonin, U46619, Pet1 knock-out mouse, vasoactive intestinal peptide, brain slices, neurogliaform cells

## Abstract

GABAergic interneurons are local integrators of cortical activity that have been reported to be involved in the control of cerebral blood flow (CBF) through their ability to produce vasoactive molecules and their rich innervation of neighboring blood vessels. They form a highly diverse population among which the serotonin 5-hydroxytryptamine 3A receptor (5-HT_3A_)-expressing interneurons share a common developmental origin, in addition to the responsiveness to serotonergic ascending pathway. We have recently shown that these neurons regroup two distinct subpopulations within the somatosensory cortex: Neuropeptide Y (NPY)-expressing interneurons, displaying morphological properties similar to those of neurogliaform cells and Vasoactive Intestinal Peptide (VIP)-expressing bipolar/bitufted interneurons. The aim of the present study was to determine the role of these neuronal populations in the control of vascular tone by monitoring blood vessels diameter changes, using infrared videomicroscopy in mouse neocortical slices. Bath applications of 1-(3-Chlorophenyl)biguanide hydrochloride (mCPBG), a 5-HT_3_R agonist, induced both constrictions (30%) and dilations (70%) of penetrating arterioles within supragranular layers. All vasoconstrictions were abolished in the presence of the NPY receptor antagonist (BIBP 3226), suggesting that they were elicited by NPY release. Vasodilations persisted in the presence of the VIP receptor antagonist VPAC1 (PG-97-269), whereas they were blocked in the presence of the neuronal Nitric Oxide (NO) Synthase (nNOS) inhibitor, L-NNA. Altogether, these results strongly suggest that activation of neocortical 5-HT_3A_-expressing interneurons by serotoninergic input could induces NO mediated vasodilatations and NPY mediated vasoconstrictions.

## Introduction

Within the cerebral cortex, different types of GABAergic inhibitory interneurons have been described according to their distinctive morphological, molecular, and electrophysiological characteristics (Cauli et al., 1997; Markram et al., 2004; Vitalis and Rossier, 2011). Although the final classification scheme of cortical interneurons is still a matter of debate (Ascoli et al., 2008), data from *in vitro* and *in vivo* experiments tend to demonstrate that distinct subpopulations of inhibitory interneurons exert specific functional roles in the integrative processes of the cortical network (Whittington and Traub, 2003; Markram et al., 2004; Fanselow and Connors, 2010; Gentet et al., 2010; Mendez and Bacci, 2011). Furthermore, some GABAergic interneurons have been reported recently to be involved in the control of cerebral blood flow (CBF) through their ability to express and release vasoactive molecules (Cauli et al., 2004; Cauli and Hamel, 2010). However, further characterization of these vasoactive interneurons subpopulations remains to be established.

Interestingly, the robust cortical serotoninergic innervation from raphe nuclei (Reinhard et al., 1979; Steinbusch, 1981; Tork, 1990), which modulate cortical activity (Takeuchi and Sano, 1984; Papadopoulos et al., 1987; DeFelipe et al., 1991) and CBF (Rapport et al., 1948; Cohen et al., 1996; Riad et al., 1998), preferentially targets inhibitory interneurons (DeFelipe et al., 1991; Smiley and Goldman-Rakic, 1996; Paspalas and Papadopoulos, 2001). However, the processes by which 5-hydroxytryptamine (serotonin, 5-HT) acts on the cortical network and CBF are complex and deserve to be further understood. Indeed, responses to 5-HT seem to depend upon the nature of the receptors involved, and the recruited neuronal populations (Underwood et al., 1992; Cohen et al., 1996; Foehring et al., 2002).

Serotonin can notably induce a fast excitation of specific interneuron subpopulations through the activation of the 5-hydroxytryptamine 3A receptor (5-HT_3A_) (Ferezou et al., 2002; Lee et al., 2010) which is the only ionotropic serotonergic receptor (Barnes and Sharp, 1999; Chameau and van Hooft, 2006). In the mouse primary somatosensory cortex, the 5-HT_3A_ receptor is expressed by two distinct types of interneurons (Vucurovic et al., 2010). The first one was characterized by a bipolar/bitufted morphology, an adaptative or bursting firing behavior and the frequent expression of the vasoactive intestinal peptide (VIP), reported to be a vasodilator in the cerebral cortex (McCulloch and Edvinsson, 1980; Yaksh et al., 1987; Dacey et al., 1988), whereas the second population of interneurons includes neurogliaform like regular spiking neurons and therefore frequently expressed the neuropeptide Y (NPY), a potent vasoconstrictor (Dacey et al., 1988; Abounader et al., 1995; Cauli et al., 2004). In rat neocortical slices, it has been shown that electrical stimulation of a single VIP- or NOS/NPY-expressing interneuron was able to induce a dilation of nearby microvessels, probably by releasing vasoactive molecules. Additionally, direct perfusion of VIP or NO donor onto cortical slices dilated blood vessels, whereas perfusion of NPY induced vasoconstrictions (Cauli et al., 2004).

In the present study, we investigated how the pharmacological activation of 5-HT_3A_-expressing interneurons can induce blood vessel diameter changes by means of infrared videomicroscopy on mice cortical slices. We find that activation of 5-HT_3A_-expressing interneurons mostly induced vasodilations mediated by NO release and also, but less frequently, vasoconstrictions through NPY release. Our results show that these interneurons are strategically positioned to transmute incoming neuronal afferent signals into vascular responses.

## Materials and methods

### Animals and surgery

Animal procedures were conducted in strict compliance with approved institutional protocols and in accordance with the provisions for animal care and use described in the *European Communities Council directive of 24 November 1986 (86-16-09/EEC)*.

C57Bl6J mice (14–21 days old; Charles River) were used for vascular reactivity. All animals were housed in a temperature-controlled (21–25°C) room under daylight conditions. They arrived in the laboratory at least 1 week before initiating experiments to acclimate to their new environment.

Immunohistochemistry was performed on a transgenic mouse line expressing the enhanced green fluorescent protein (GFP) under the control of the 5-HT_3A_ promoter (5-HT3A:GFP). This line, obtained by using modified bacterial artificial chromosomes (BACs) was provided by the GENSAT Consortium (Rockefeller University-GENSAT Consortium; (Heintz, 2004) and maintained under the Swiss genetic background by breeding heterozygous mice. The selective expression of GFP in 5-HT_3_ expressing neurons has been previously controlled in the cortex of these mice (Lee et al., 2010; Vucurovic et al., 2010).

The Pet1 knock-out mouse line (gift from Evan Deneris, Case Western Reserve University, Cleveland, OH) was maintained on a C57BL6 genetic background. Heterozygous Pet1^+/−^ females were mated with Pet1^+/−^ or Pet1^−/−^ males to produce mixed litters. Genotyping of the progeny was done by PCR analysis of tail DNA as described previously (Hendricks et al., 2003).

### Immunohistochemistry

Neuronal populations expressing 5-HT_3A_:GFP were analyzed at postnatal day P18–P25 (*n* = 6). Animals were deeply anesthetized with an intraperitoneal (IP) injection of pentobarbital (150 mg/kg body weight) and perfused transcardially with 4% paraformaldehyde (PFA). Brains were cryoprotected in 30% sucrose and cut on a freezing microtome (35 μm). For immunofluorescence, sections were incubated overnight at 4°C with the following antibodies diluted in phosphate buffer (PS) saline (PBS): chicken anti-GFP (1:1000, Molecular Probes) and rabbit anti-NPY (1:500, Amersham), rabbit anti-VIP (1:800, Incstar) or rabbit anti-nNOS (1:400, Santa-Cruz). After washing in PBS, sections were incubated in alexa 568 conjugated goat anti-rabbit and alexa 488 conjugated goat anti-chicken antibodies (1:300; Molecular Probes).

Sections were rinsed in PB, mounted in Vectashield (Vector) containing 4′,6′-diamidino-2-phenylindole (DAPI) and were observed with a fluorescent microscope (Leica, DMR). Images were acquired with a Coolsnap camera (Photometrics). Quantifications of GFP:5-HT^+^_3A_ and NOS^+^, NPY^+^, or VIP^+^ cells were performed at the level of the primary somatosensory cortex, in 500 μm-wide cortical strips (data are expressed as percentages). Three adjacent sections of at least five animals were used.

The estimation of the neuronal density at specific distances from the closest penetrating blood vessel was performed on coronal brain slices (60 μm thick) that were fixed by immersion overnight in PFA. Slices were then rinsed in PBS prior immunolabeling. For immunofluorescence, sections were incubated sequentially overnight at 4°C with the following antibodies diluted in PBS: rabbit anti-GFP (1:1000, Molecular Probes) and goat anti-collagen IV (1:400, Millipore). After washing in PBS, sections were incubated in CY3 conjugated rabbit anti-goat and alexa 488 conjugated chicken anti-rabbit antibodies (1:300; Molecular Probes). Sections were rinsed in PB, mounted in Vectashield (Vector) containing 4′,6′-diamidino-2-phenylindole (DAPI) and were observed with a fluorescent microscope (Zeiss). Images were acquired at the level of the somatosensory cortex with a Zeiss microscope equiped with an AxioCam MRm CDC camera (Zeiss) and three adjacent sections of four animals were used. When two penetrating blood vessels were identified the distance of GFP:5-HT^+^_3A_ cells within the region defined by these two blood vessels was determined. The distance from the closest penetrating blood vessel was calculated using Image J software. The distance from the closest blood vessel and the center of two adjacent penetrating blood vessels was subsequentially subdivided in four equal parts. The percentages of 5-HT_3A_:GFP^+^ cells located within these four radial bins were then calculated.

Some captions were also taken with a Nikon confocal system and maximal projections were used for illustration.

### Preparation of acute cortical slices

Mice were rapidly decapitated and brains were quickly removed and placed into cold (~4°C) slicing solution containing (in mM): 110 choline chloride, 11.6 Na-ascorbate, 7 MgCl_2_, 2.5 KCl, 1.25 NaH_2_PO_4_, and 0.5 CaCl_2_, continuously bubbled with 95% O_2_–5% CO_2_. Coronal brain slices (300 μm thick) containing the primary somatosensory cortex were cut with a vibratome (VT1200S; Leica), and transferred to a holding chamber enclosing artificial cerebrospinal fluid (ACSF) containing (in mM): 126 NaCl, 2.5 KCl, 1.25 NaH_2_PO_4_, 2 CaCl_2_, 1 MgCl_2_, 26 NaHCO_3_, 20 glucose and 1 kynurenic acid (a nonspecific glutamate receptor antagonist, Sigma), constantly oxygenated (95% O_2_–5% CO_2_), kept 30 min at 35°C and then held at room temperature. Individual slices were next placed in a submerged recording chamber kept at 32°C and perfused (1.5 ml/min) with oxygenated ACSF (in the absence of kynurenic acid). Blood vessels were visualized using infrared videomicroscopy with Dodt gradient contrast optics (Luigs and Neumann) mounted on an upright microscope (Zeiss) equipped with a CDD camera (Hamamatsu).

### Drugs

The 5-HT_3_ receptor selective agonist (1-(3-Chlorophenyl)biguanide hydrochloride, mCPBG, 100 μM, Sigma) was used to stimulate specifically 5-HT_3_-expressing interneurons. To block neuronal synaptic transmission, mCPBG was applied in the presence and after 10 min application of tetrodotoxin (TTX; 1 μM; Latoxan). To block the nNOS, slices were treated for at least 1 h with an irreversible inhibitor of constitutive nitric oxide synthase (nNOS) and a reversible inhibitor of inducible nitric oxide synthase (iNOS), Nω-Nitro-L-arginine (L-NNA; 100 μM; Sigma). NPY Y1 receptors were blocked with the selective antagonist N2-(Diphenylacetyl)-N-[(4-hydroxyphenyl) methyl]-D-arginine amide (BIBP 3226; 100 μM; Sigma). VIP type 1 (VPAC1) receptors of VIP were blocked with the high-affinity, selective antagonist PG-97-269 (100 nM; Biochem, Shanghai). The thromboxane A_2_ receptors agonist (9,11-dideoxy-11a,9a-epoxymethanoprostaglandin F2α, U46619, 5 nM, Sigma) was used to pre-constrict the blood vessels.

### Vascular reactivity

Arterioles remaining in focal plane, exhibiting a well-defined luminal diameter (10–20 μm) and located in the supragranular layers of the somatosensory cortex were selected for vascular reactivity. Images of arterioles were acquired every 15 s (Media Cybernetics). The eventual drift of the images along the time of the recording was corrected on-line for the z-drift and off-line for *x* and *y* directions using Image Pro Plus 7.0. Manual replacement of the images to minimize the differences between two consecutive frames was realized by using a subtraction tool from Image Pro Plus and luminal diameters were quantified at different locations along the blood vessel using custom written routines running within IgorPro (WaveMetrics) to determine the location that moved the most.

Control baselines were determined for 5 min at the start of the recording. Unresponsive blood vessels or vessels with unstable baseline were discarded from the analysis. Vessels were considered unstable when their diameter moved more than 5% during the control baseline. Only one vessel per slice was recorded.

As blood vessels in the slice preparation lack intraluminal flow and pressure (Sagher et al., 1993; Cauli et al., 2004), vasomotor movements were detected in vessels pre-constricted for 10 min with the thromboxane agonist U46619 (5 nM), which was applied throughout the experiment. Only blood vessels that presented a diameter reduction of at least 10% were kept for analyses. The vasoconstriction of blood vessels induced by U46619 followed an exponential progression along the time. This exponential drifting contraction was fitted and subtracted from the recordings using IgorPro.

Maximal vasomotor responses were then expressed as percentages of the mean baseline diameter, which is the averaged diameter measured during the control period of one minute, after correction for the pre-constriction and just before the mCPBG application.

The maximal response diameter was averaged between the fifth and sixth minute after the onset of the mCPBG application for vasodilations and between the fourth and the fifth minute for vasoconstrictions.

### Statistical analyses

Data were expressed as mean ± standard error of the mean (SEM). A paired *t*-test was used to determine the statistical significance of vasomotor responses to mCPBG application under the same conditions and a *t*-test was used to determine the statistical significance of response amplitudes between different set of experiments.

## Results

### Distribution of 5-HT_3A_-expressing neurons and co-expression of vasoactive molecules in transgenic 5-HT_3A_:GFP mice

**Figure 1:**
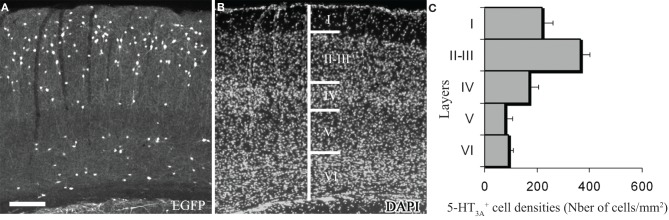
**Expression of 5-HT_3A_ within the somatosensory cortex. (A,B)** Coronal section of a 5-HT_3A_:GFP mouse counterstained with DAPI showing the preferential location of 5-HT_3A_-expressing cells in supragranular layers. **(C)** Density of 5-HT_3A_-expressing cells from transgenic 5-HT_3A_:GFP mice in the different layers of the primary somatosensory cortex. Scale bar: 250 μm.

In order to determine the laminar distribution of 5-HT_3_-expressing interneurons and the vasoactive molecules that they express, we used a reporter mouse strain in which the enhanced GFP expression is under the control of the 5-HT_3A_ promoter (Heintz, 2001). This 5-HT_3A_:GFP mouse line provided by GENSAT shows similar distribution of cortical 5-HT_3A_:GFP^+^ neurons, compared to the expression pattern of 5-HT_3A_ transcripts observed in wild type animals (Vucurovic et al., 2010). The 5-HT_3_-expressing interneurons are distributed in all cortical layers (Figure 1), with a higher density in supragranular layers.

**Figure 2:**
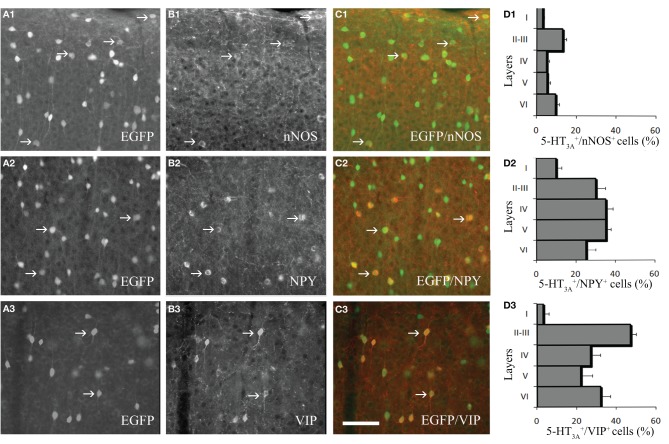
**Expression of vasoactive molecules in 5-HT_3A_-expressing interneurons. (A1–3)** Immunodetection of GFP-expressing neurons within the primary somatosensory cortex of 5-HT_3A_:GFP^+^ mice. **(B1–3)** Immunodetection of nNOS (B1), NPY (B2) and VIP (B3) within the same areas as in (A). **(C1–3)** Overlays showing the colocalization of GFP with nNOS (C1), NPY (C2), or VIP (C3). **(D1–3)**, Density of 5-HT_3A_-expressing cells colocalized with nNOS (D1), NPY (D2), or VIP (D3) in the different layers of the somatosensory cortex. Examples of co-labeled neurons are pointed by arrows. Scale bar: 65 μm.

We next performed immunohistochemical analyses to assess the expression of vasoactive molecules in 5-HT_3A_-expressing interneurons. Within the primary somatosensory cortex, 5-HT_3A_:GFP^+^ cells frequently co-expressed NPY (31 ± 4.3%), VIP (30.2 ± 5.2% or nNOS (9.8 ± 0.9%; Figure 2). These nNOS^+^/GFP^+^ cells correspond to 34.4 ± 0.9% of type II nNOS cells (see Perrenoud et al., 2012), whereas no type I nNOS cell were found to express 5-HT_3_.

The proportion of cells in which GFP was co-detected with one of the three markers is layer dependent (Figure 2D). Indeed, VIP^+^/GFP^+^ interneurons were preferentially located in layers II-III, while nNOS^+^/GFP^+^ and NPY^+^/GFP^+^ interneurons were distributed in all cortical layers. These distributions correspond to those recently reported by Lee and collaborators for the NPY and VIP (Lee et al., 2010).

**Figure 3:**
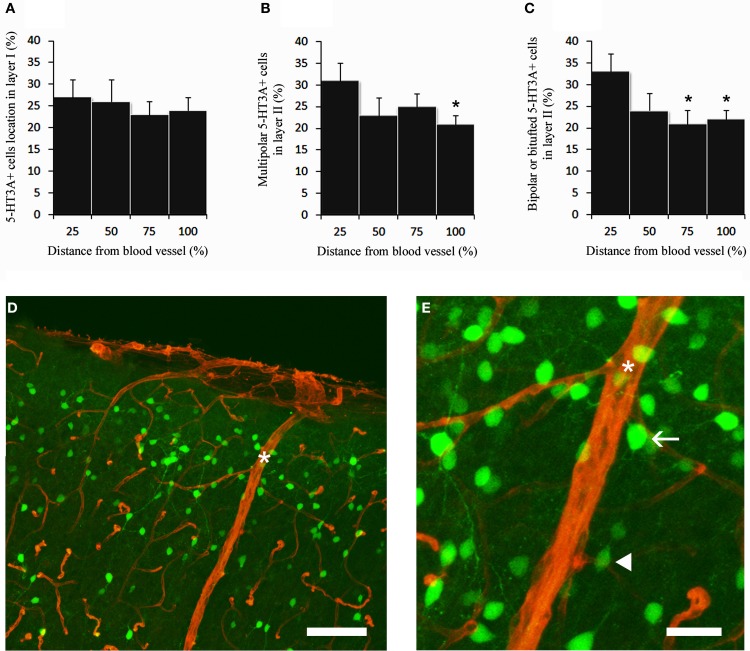
**5-HT_3A_:GFP^+^ neurons in relation with the closest large penetrating blood vessel. (A)** In layer I, 5-HT_3A_:GFP^+^ neurons do not show specific preferences to be located close to large penetrating blood vessels. **(B,C)** By contrast in layer II multipolar and bipolar/bitufted 5-HT_3A_:GFP^+^ neurons are preferentially positioned in the vicinity of penetrating blood vessels. **(D)** Confocal picture of a coronal section taken at the level of the supragranular layers of the somatosensory cortex showing the distribution of 5-HT_3A_:GFP^+^ neurons (green) in relation with blood vessels stained by collagen IV (red). Large penetrating blood vessel is indicated by a star. **(E)** Higher power view of the caption shown in **(D)**. The arrow points to a multipolar 5-HT_3A_:GFP^+^ neurons displaying a large soma. The filled arrowhead points to a bipolar 5-HT_3A_:GFP^+^ neurons displaying a fusiform shape. Scale bars: (D) 130 μm; (E) 35 μm.

We then determined the relative distribution of 5-HT_3A_:GFP^+^ neurons in relation with the closest large penetrating blood vessel identified on a coronal section at the level of the somatosensory cortex (17–32 μm, Figure 3). This was done in layer I and in layer II were subsequent analyses were performed. In layer I the distribution of 5-HT_3A_:GFP^+^ cells was homogenous between two penetrating blood vessels (Figure 3A). By contrast, in layer II, both multipolar 5-HT_3A_:GFP^+^ neurons (Figure 3B) and bipolar 5-HT_3A_:GFP^+^ neurons (Figure 3C) showed an non-uniform distributions as the densities of 5-HT_3A_:GFP^+^ neurons were higher closest two the penetrating blood vessel (0–25% away from the closest blood vessel) then away from it (75–100% for multipolar 5-HT_3A_:GFP^+^ neurons, *P* < 0.05 and 50–100% for bipolar 5-HT_3A_:GFP^+^ neurons, *P* < 0.01).

### Pharmacological stimulation of 5-HT_3A_-expressing interneurons in cortical slices induces vasomotor movements recorded by infrared videomicroscopy

**Figure 4:**
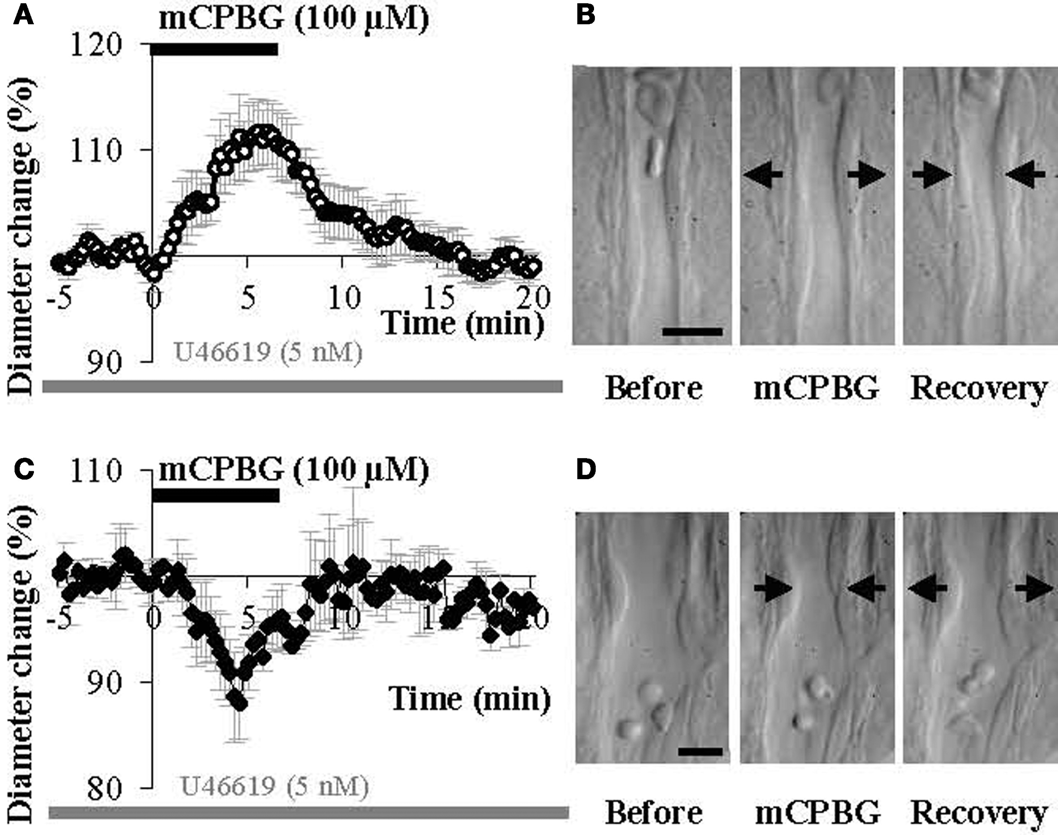
**mCPBG induces both vasodilations and vasoconstrictions in somatosensory cortical slices. (A)** Mean vascular dilation (*n* = 9) induced by mCPBG (100 μM). **(B)** Infrared images of a penetrating blood vessel that reversibly dilated in response to bath application of mCPBG (100 μM). Arrows indicate region of high vascular reactivity. **(C)** Mean vascular constriction (*n* = 4) induced by mCPBG (100 μM). **(D)** Infrared images of a penetrating blood vessel that reversibly constricted in response to bath application of mCPBG (100 μM). Scale bar: 10 μm.

The stimulation of GABAergic interneurons can induce vasomotricity via the production of vasoactive substances such as VIP, NPY, or nitric oxide (NO), a highly diffusible gas which is known to be a potent vasodilator (Estrada and DeFelipe, 1998; Cauli et al., 2004; Rancillac et al., 2006). Here we investigated the functional role of 5-HT_3_-expressing interneurons in the control of vascular tone within the supragranular layers of the cortex. In this aim, we applied a 5-HT_3_R selective agonist mCPBG (100 μM) for 6 min, directly onto acute slices while recording associated blood vessel movements using infrared videomicroscopy. We focused our study on penetrating arterioles that are known to be of prime importance in feeding deeply located microvessels and neurons (Nishimura et al., 2007). Well defined arterioles of supragranular layers were therefore selected for quantitative analyses. As blood vessels in the slice preparation lack intraluminal flow and pressure (Sagher et al., 1993; Cauli et al., 2004), vasomotor changes were detected in pre-constricted arterioles using the thromboxane agonist U46619 (5 nM) throughout the experiment. When bath applied, mCPBG (100 μM) induced either reversible vasodilations (111.11 ± 3.03% over baseline, *n* = 9/17; *P* < 0.01; Figures 4A and B) or reversible vasoconstrictions (90.04 ± 3.15% over baseline, *n* = 4/17; *P* < 0.05; Figures 4C and D).

### Vasodilations are mainly mediated by no whereas vasoconstrictions are due to NPY release

**Figure 5:**
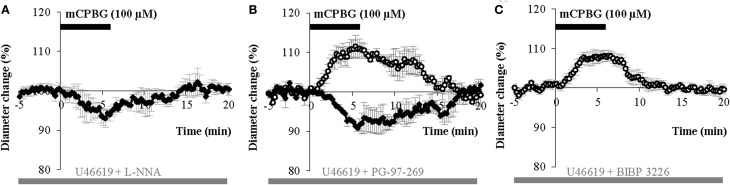
**mCPBG induced vasodilations are mediated by NO while constrictions are mediated by NPY. (A)** Mean vasoconstriction (*n* = 4) induced by mCPBG (100 μM) in the presence of nNOS inhibitor L-NNA (100 μM) and the preconstricting agent U46619 (5 nM). **(B)** Mean vascular dilation (*n* = 4; white circle) and mean vascular constriction (*n* = 2; black diamond) induced by mCPBG (100 μM) in the presence of the VIP receptor VPAC1 antagonist PG-97-269 (100 nM) and U46619 (5 nM). **(C)** Vasodilation (*n* = 9) induced by mCPBG (100 μM) in the presence of the NPY Y1 receptor antagonist BIBP 3226 (1 μM) and U46619 (5 nM).

**Figure 6:**
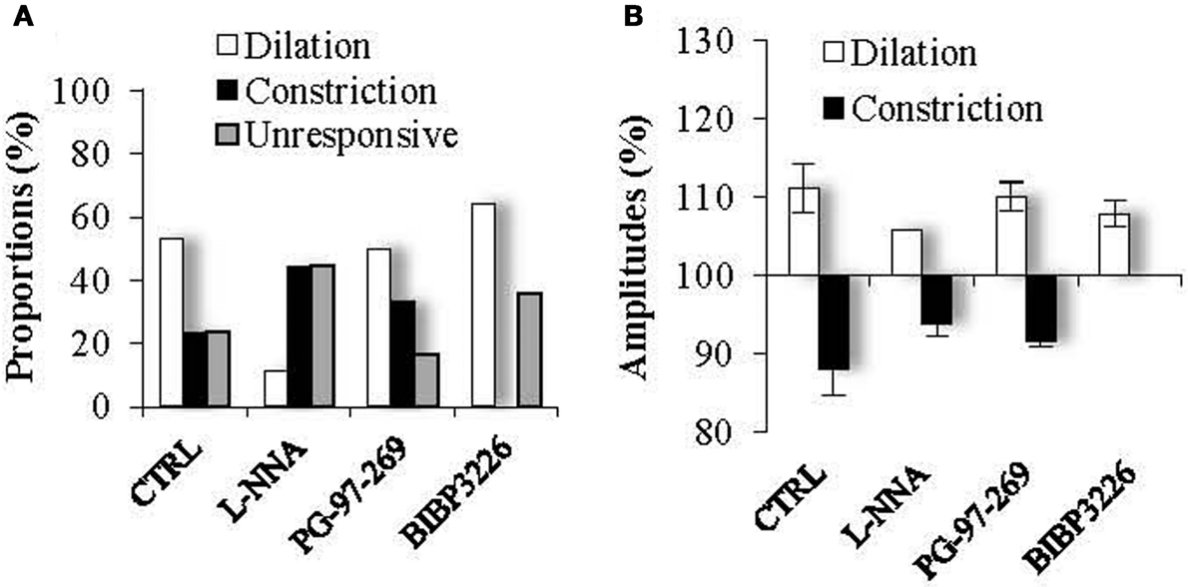
**Proportions (A) and amplitudes (B) of dilating or constricting blood vessels in control condition (CTRL) and in the presence of the pharmacological blockers used in Figure 5.** Note that in presence of the nNOS inhibitor, L-NNA, as in the presence the Y1 NPY receptor antagonist, BIBP 3226, the occurrence of dilations *versus* contractions was reduced or favored respectively, without impacting their amplitudes. On the opposite, in the presence of the VPAC1 receptor antagonist of VIP, PG-97-269, no changes were observed compared to control conditions.

We hypothesized that mCPBG induced vasodilation could be due to NO and/or VIP release by 5-HT_3A_R-interneurons, whereas vasoconstriction could be caused by NPY release. To determine the molecular events underlying vasomotor changes, we successively blocked different possible mechanisms. Lowering basal NO levels by treatment with the constitutive nNOS inhibitor L-NNA (100 μM) strongly reduced the proportion of vasodilations observed in response to mCPBG applications from 69% in control conditions to 11% (*n* = 1/9) and favored vasoconstrictions from 31% in control condition to 80% (93.83 ± 1.60 of baseline diameter; *n* = 4/9, *P* < 0.01) (Figures 5A and 6).

These results suggest that mCPBG-induced vasodilations are mostly mediated by NO release. The remaining vasodilation observed in the presence of L-NNA could be mediated by VIP release. However, in the presence of the VIP receptor VPAC1 antagonist, PG-97-269 (100 nM), mCPBG application induced 60% of vasodilations (110.01 ± 1.87%; *n* = 3/6; *P* < 0.05) and 40% of vasoconstrictions (92.04 ± 0.61; *n* = 2/6; *P* < 0.05) of similar proportions and amplitudes compared to control conditions (Figures 5B and 6). Then, to determine the molecular pathway underlying vasoconstrictions, treatment of mCPBG was reproduced in the presence of NPY Y1 receptor antagonist (BIBP 3226, 1 μM). Indeed, vasoconstrictions mediated by NPY are known to be mediated by smooth muscle NPY Y1 vascular receptor (Abounader et al., 1999). Under BIBP 3226, constrictions where blocked and only dilations (108.28 ± 1.62%; *n* = 9/14; *P* < 0.01) could be recorded (Figures 5C and 6). Amplitudes of vasomotor responses under these different conditions were not statistically different from control condition (Figure 6B). Altogether, these data strongly suggest that pharmacological stimulations of 5-HT_3A_-expressing interneurons mainly induce vasodilations through NO release, whereas they induce to a less extensive vasoconstrictions through NPY release and activation of its Y1 receptor.

### TTX-insensitive peptidergic and no release

**Figure 7:**
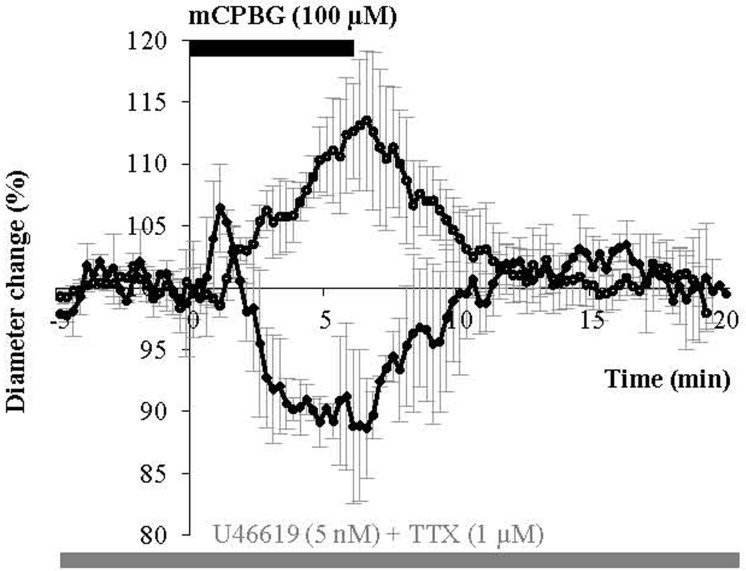
**mCPBG induces TTX-insensitive vasodilations and vasoconstrictions.** Mean vascular dilation (*n* = 5) and constriction (*n* = 2) induced by mCPBG (100 μM) in the presence of TTX (1 μM) and U46619 (5 nM).

It is assumed that peptidergic release requires repetitive action potentials at high frequencies (Zupanc, 1996; Ludwig and Pittman, 2003; Baraban and Tallent, 2004) and that nNOS activation depends on Ca^2+^ entry (Garthwaite, 2008). Therefore, we tested mCPBG stimulation in the presence of tetrodotoxin (TTX, 1 μM) to block action potential generation and propagation. However, this treatment failed to prevent mCPBG-induced vasodilations (111.45 ± 4.81%; *n* = 5/10, *P* < 0.05) or vasoconstrictions (90.04 ± 2.1%; *n* = 2/10, *P* < 0.05), (Figure 7). This suggests that the vasomotor changes observed in response to 5-HT_3_R activation may depend on the Ca^2+^ influx through 5-HT_3_R rather than on action potential generation.

### Origin of 5-HT_3_R induced no release

**Figure 8:**
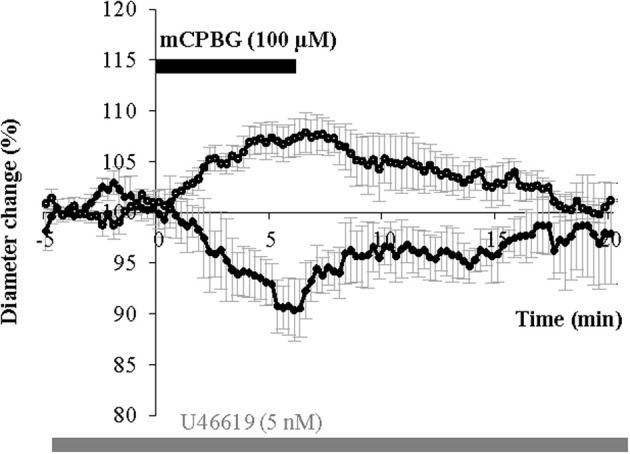
**mCPBG induces vasodilations and vasoconstrictions in Pet1^−/−^ mice.** Mean vasodilation (*n* = 6) and vasoconstriction (*n* = 3) induced by mCPBG (100 μM) in Pet1^−/−^ mice.

In the neocortex the 5-HT_3A_R is exclusively located on GABAergic interneurons (Morales and Bloom, 1997; Ferezou et al., 2002; Chameau and van Hooft, 2006; Vucurovic et al., 2010). However, 5-HT inputs from the raphe also express presynaptic 5-HT_3A_Rs regulating neurotransmitter release (Jackson and Yakel, 1995; Roerig et al., 1997; Nayak et al., 1999). As the presence of NOS in 5-HT-containing axons from dorsal raphe has been already reported in the somatosensory cortex (Simpson et al., 2003) the pharmacological stimulation of 5-HT_3_Rs realized in this study could also have induced NO release from the NOS-containing fibers originating from raphe nuclei. In order to evaluate the contribution of such a NO release in our experiments, we used Pet1^−/−^ (Pheochromocytoma 12 ETS factor-1) knock-out mice, in which raphe projections to the somatosensory cortex were found to be strongly reduced (Hendricks et al., 1999; Liu et al., 2010). In this mutant mice, we found that pharmacological stimulation of 5-HT_3_R could still induce vasodilations (107.07 ± 1.67%; *n* = 6/11, *P* < 0.01) or vasoconstrictions (93.45 ± 2.60%; *n* = 3/11, *P* < 0.05), (Figure 8). These vasomotor changes were not significantly different than those induced in control condition neither in amplitude nor in proportion (from 69 to 67% for vasodilations and from 31 to 33% for vasoconstrictions), suggesting a postsynaptic origin of NO release.

## Discussion

**Figure 9:**
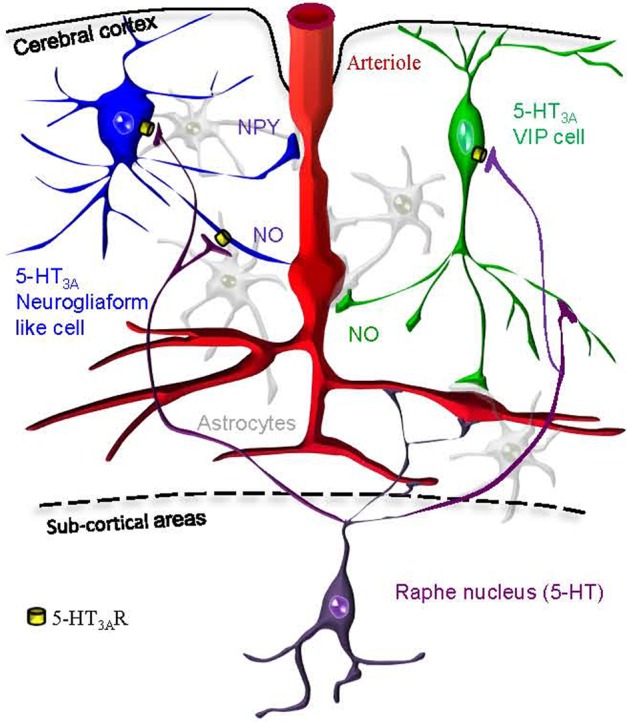
**Hypothetical schematic representation of the 5-HT_3A_R induction of neurovascular coupling within the somatosensory cortex.** We find that pharmacological stimulation of 5-HT_3A_-expressing neurogliaform like cells (in blue) or VIP cells (in green) could either lead to vasodilation via NO release or vasoconstriction via NPY release. Cortical penetrating arterioles (in red) are also directly innervated by serotoninergic fibers originating from raphe nuclei (in purple). Astrocytes are shown in gray.

5-HT_3A_-expressing interneurons are composed of two different populations: neurogliaform like interneurons expressing NPY and bipolar/bitufted interneurons expressing VIP that are likely candidates to control cortical blood vessels tonus via neuronally derived vasoactive messengers. In this article we show that in supragranular layers of the mouse somatosensory cortex, pharmacological activation of 5-HT_3_-expressing interneurons can release of NO to dilate, or NPY to constrict arterioles (Figure 9).

### 5-HT_3A_R activation induces vascular responses through the activation of specific GABAergic interneurons subpopulations

The mechanisms underlying the vasoactive action of serotonin in the cerebral cortex are not completely understood at present time. Our results demonstrate that the selective activation of the ionotropic 5-HT receptor, 5-HT_3_R, induces a complex vascular response including both local vasoconstrictions and vasodilations.

These vasomotor changes are not mediated by the presynaptic 5-HT_3_R activation of serotonergic axons originating from raphe nuclei that could potentially release vasoactive substances. Indeed, they were also observed in Pet-1^−/−^ knock-out mice, which display a drastic depletion of cortical serotoninergic fibers (Hendricks et al., 1999; Liu et al., 2010; Kiyasova et al., 2011). Conversely, our hypothesis is that a specific activation of local 5-HT_3A_-expressing GABAergic interneurons is responsible for the observed vasomotor responses.

Indeed, it has been reported that 5-HT_3A_ expression is confined to GABAergic interneurons in both rat and primate cerebral cortex (Morales and Bloom, 1997; Jakab and Goldman-Rakic, 2000; Ferezou et al., 2002). This has been recently confirmed in the mouse somatosensory cortex since neither the pyramidal cell marker Satb2 nor the oligodendrocyte marker Olig2 could be detected in 5-HT_3A_-expressing cells (Lee et al., 2010).

5-HT_3A_-expressing interneurons constitutes nearly 30% of the total interneuronal population (Rudy et al., 2011), which accounts for ~15% of the overall cortical neurons in rodents (Beaulieu, 1993; Gabbott et al., 1997). Using an unsupervised cluster analysis based on 28 electrophysiological parameters, we have shown in a previous study (Vucurovic et al., 2010) that 5-HT_3A_-expressing interneurons are segregated into two distinct subpopulations within the somatosensory cortex. The first one was characterized by the frequent expression of the NPY (87%) and a multipolar morphology, while the second one expressed frequently the VIP (73%), and presented a bipolar somatodendritic shape. Subsequently, in an article published in 2010, Lee et al. have suggested, based on immunohistochemical analyses and electrophysiological recordings, a much broader diversity of the 5-HT_3A_-expressing neuronal population. Indeed, analyzing the intrinsic membrane properties and the morphology of these neurons, they distinguished seven types of 5-HT_3A_-expressing interneurons. Although our separation of these neurons in two subpopulations could therefore appear restrictive; we consider it is meaningful since it relies on a solid statistical classification analysis (unsupervised clustering). However, we do not exclude the existence of some diversity within these two groups.

In the present study, we analyzed the laminar distribution of these neuronal populations. We observed that the cell bodies of more than two-thirds of these interneurons were positioned within superficial layers (L1, 13 ± 0.9%; L2/3, 51 ± 1.2%) in agreement with what was recently described (Lee et al., 2010; Vucurovic et al., 2010). We further revealed that the nNOS is also expressed by a subset of 5-HT_3A_-expressing interneurons. These neurons corresponded to type II NOS^+^ cells, since they were smaller than type I and weakly immunohistochemically-stained for nNOS (Yan et al., 1996; Cho et al., 2010; Perrenoud et al., 2012). Thus, the distribution and the expression of vasoactive substances within 5-HT_3A_-expressing interneurons put them in a unique position to induce a fast and effective modulation of vascular tone in response to 5-HT.

### Dual role of 5-HT_3A_-expressing interneurons on vasomotor control

In line with our finding that 5-HT_3A_-expressing interneurons produce several vasoactive substances, our results indicate that their activation induces a complex vascular response. Indeed the selective 5-HT_3_R agonist mCPBG induced either constrictions (30%) or dilations (70%) of penetrating arterioles within supragranular layers. All vasoconstrictions were abolished in the presence of the NPY receptor antagonist (BIBP 3226), suggesting that they were elicited by NPY release. Conversely, mCPBG-induced dilations were blocked in the presence of a nNOS inhibitor suggesting that NO is the predominant messenger inducing the vasodilation, in line previous studies performed in the cerebellum (Yang et al., 1999, 2000; Rancillac et al., 2006) and in the cortex (Estrada and DeFelipe, 1998; Lovick et al., 1999; Brown et al., 2000; Tong and Hamel, 2000; Cauli et al., 2004; Liu et al., 2008; Rancillac et al., 2012). Interestingly, a recent study on epileptic seizures has revealed that 5-HT_3_ activation can stimulate NO synthesis (Gholipour et al., 2010).

The NPY-mediated constrictions confirm and extend prior studies implicating NPY in vasoconstrictions (Dacey et al., 1988; Abounader et al., 1995; Cauli et al., 2004) and are likely to be caused by the activation of NPY/5-HT_3A_-expressing neurogliaform-like cells (Lee et al., 2010; Vucurovic et al., 2010).

On the other hand, it has been shown that neurogliaform cells substantially express nNOS (Karagiannis et al., 2009; Kubota et al., 2011; Perrenoud et al., 2012) and are thus well positioned to mediate NO-induced vasodilations. In addition, we have recently found that a significant proportion of nNOS type II cells express VIP (Perrenoud et al., 2012). Therefore, a subset of VIP/5-HT_3_-expressing cells could also release NO following 5-HT_3A_R stimulation (Figure 9).

5-HT_3A_-nNOS-expressing interneurons could also form a small third class of 5-HT_3A_-expressing interneurons that express neither NPY nor VIP. Although this population has not been isolated with the cluster analysis of these interneurons (Vucurovic et al., 2010), further triple immunolabeling would be necessary to assess this possibility.

Unexpectedly, although VIP is present in 5-HT_3A_-expressing interneurons, the mCPBG-induced dilations persisted in the presence of the VIP receptor VPAC1 antagonist, suggesting that VIP release acting on VPAC1 receptors is not involved in this response. This was surprising since the VPAC1 receptor is a postsynaptic receptor predominantly and uniformly expressed by smooth muscle cells in cerebral arteries and arterioles (Fahrenkrug et al., 2000) and that direct perfusion of VIP as well as electrical stimulation of a single VIP-expressing interneuron have been reported to dilate microvessels onto rat slices (Cauli et al., 2004). Moreover, both light and electron microscopy have revealed that some VIP^+^ neurons are closely associated with blood vessels, providing a neuroanatomic substrate for the role of VIP in the regulation of cerebral blood circulation in the rat cerebral cortex (Eckenstein and Baughman, 1984; Chédotal et al., 1994a,b; Fahrenkrug et al., 2000).

In the presence of a nNOS inhibitor, one dilation out of nine tested blood vessels was still observed (Figure 6A). This dilation could have been mediated by an activation of perivascular astrocytes resulting from the evoked action potential discharge of 5-HT_3_ interneurons. Indeed, astrocytes are known as cellular intermediaries that couple neuronal activity to local blood flow changes through the phospholipase A_2_ (PLA_2_)-mediated synthesis of arachidonic acid, which leads to production of prostaglandins and epoxyeicosatrienoic acids (Petzold and Murthy, 2011). However, Kitaura et al. (2007) have shown that hindpaw stimulation-induced cortical vasodilations were suppressed in mice lacking nNOS, while they were unchanged in mice lacking cytosolic phospholipase A2 alpha (cPLA2α).

Together, our observations that in the somatosensory cortex a selective 5-HT_3_R agonist induce NO mediated vasodilations confirms and extends prior studies reinforcing the central role of NO in the neurovascular coupling.

### TTX-insensitive vasomotor changes

The cellular mechanisms underlying the release of vasoactive compounds within the cortex are still poorly considered. Here we observed that the vascular responses induced by 5-HT_3A_R activation were unaffected by the bath application of TTX. This result indicates that the release of NO, NPY, and VIP by 5-HT_3_-expressing interneurons is independent of subthreshold activity, raising the question of the mode of secretion of these molecules in the neutrophil.

Studies of hypothalamic neuroendocrine cells indicate that neuropeptides are confined to large dense-core vesicles and released from dendrites and axons (Ludwig and Pittman, 2003) following high intracellular Ca^2+^ concentrations (Kits and Mansvelder, 2000). Action potentials originating at the neuronal soma trigger neuropeptide release from terminals, whereas Ca^2+^ release from intracellular stores signals dendritic peptide release (Ludwig et al., 2002). Similarly, the nNOS enzyme, which immunoreactivity spreads throughout the cytoplasm of neuronal cell bodies and their processes (Batista et al., 2001), is well known to be activated by Ca^2+^ associated with calmodulin (Bredt et al., 1991).

Therefore, if these mechanisms are conserved within the cortex, NPY and NO release should also be dependent on high intracellular Ca^2+^ concentrations influx. Since 5-HT_3A_Rs are predominantly expressed at the somatodendritic level in the cortex (Morales et al., 1996; Jakab and Goldman-Rakic, 2000), our study indicates that the application of mCPBG is likely to evoke enough intracellular Ca^2+^ influx through 5-HT_3A_R to induce Ca^2+^ release from intracellular stores and trigger NPY and NO release.

Several lines of evidence support this hypothesis. In heterologous expression systems, the Ca^2+^ permeability of the 5-HT_3_R has been reported to be enhanced by co-assembly of the 5-HT_3A_ subunit with the α-4 subunit of the nicotinic acetylcholine receptor (van Hooft et al., 1998; Sudweeks et al., 2002). The existence of functional heteromeric 5-HT_3A/_nACh_α4_ receptors has been described in CA1 hippocampal interneurons (Sudweeks et al., 2002). Lee et al. (2010), recently reported that in the mouse somatosensory cortex, like in the rat somatosensory cortex (Ferezou et al., 2002), 5-HT_3A_-expressing neurons respond to both 5-HT_3_R and nAChR agonists. Hence, heteromeric 5-HT_3/_nACh_α4_ receptors are probably present on the membrane of cortical 5-HT_3A_-expressing interneurons and potentiate the Ca^2+^ influx evoked by the mCPBG. Altogether, these results suggests that NPY and NO release were not triggered by action potential generation, but rather by direct Ca^2+^ entry through homomeric or heteromeric 5-HT_3A_R channels in response to mCPBG application, supporting the fact that TTX blocked neither NO nor neuropeptides induced vasomotor responses.

### Impact of 5-HT_3A_-expressing interneurons activation on the cortical network

*In vitro* electrophysiological studies have shown that ionotropic serotonergic receptor agonists induce a fast excitation of 5-HT_3A_-expressing interneurons through the activation of postsynaptic somatodendritic receptors (Porter et al., 1999; Zhou and Hablitz, 1999; Ferezou et al., 2002; Lee et al., 2010). This activation is likely to have inhibitory impact on the cortical network. Indeed, because supragranular interneurons innervate pyramidal cells, their activation will induce inhibitory post-synaptic currents in pyramidal neurons and thus inhibits their firing activity (Zhou and Hablitz, 1999; Moreau et al., 2010).

Nonetheless, the physiological release of 5-HT by the fibers originating from the raphe nuclei activate not only 5-HT_3A_Rs, but also metabotropic serotonin receptors subtypes that are divided into six classes (5-HT_1_R, 5-HT_2_R, and 5-HT_4_R–5-HT_7_R) (Hoyer et al., 1994; Walstab et al., 2010; Pytliak et al., 2011). The consequences of intracerebrally released 5-HT, both on the cortical network activity and on the CBF will be based on its various actions through different 5-HTRs (Cohen et al., 1996; Andrade, 2011). Indeed, 5-HT has been reported to have both inhibitory and excitatory effects on the cortical network (Krnjevic and Phillis, 1963; Reader, 1978; Waterhouse et al., 1990; Zhou and Hablitz, 1999; Puig et al., 2005), while it has a negative impact on the CBF in the neocortex through a major vasoconstrictor role of 5-HT through 5-HT_1B_ receptors (Cohen et al., 1996; Riad et al., 1998).

## Conclusion

Altogether, this study indicates that 5-HT_3A_-expressing interneurons occupy a strategic position in superficial layers to convey fast effects of serotonergic modulation, thus modulating cortical network activity and blood supply. The brainstem 5-HT pathway, in addition to its direct projections and vasomotor effects on cortical blood vessels, can use 5-HT_3A_-expressing interneurons to control and adapt CBF. These results suggest that blood flow could be enhanced prior to the onset of any metabolic deficit, reinforcing the “neurogenic” hypothesis of the neurovascular coupling versus the “metabolic” one (Rossier, 2009).

### Conflict of interest statement

The authors declare that the research was conducted in the absence of any commercial or financial relationships that could be construed as a potential conflict of interest.

## References

[B1] Abounader R. Elhusseiny A. Cohen Z. Olivier A. Stanimirovic D. Quirion R. Hamel E. (1999). Expression of neuropeptide Y receptors mRNA and protein in human brain vessels and cerebromicrovascular cells in culture. J. Cereb. Blood Flow Metab. 19, 155–163 10.1097/00004647-199902000-00007 10027771

[B2] Abounader R. Villemure J. G. Hamel E. (1995). Characterization of neuropeptide Y (NPY) receptors in human cerebral arteries with selective agonists and the new Y1 antagonist BIBP 3226. Br. J. Pharmacol. 116, 2245–2250 8564255 10.1111/j.1476-5381.1995.tb15060.x PMC1908978

[B3] Andrade R. (2011). Serotonergic regulation of neuronal excitability in the prefrontal cortex. Neuropharmacology 61, 382–386 10.1016/j.neuropharm.2011.01.015 21251917 PMC3110517

[B4] Ascoli G. A. Alonso-Nanclares L. Anderson S. A. Barrionuevo G. Benavides-Piccione R. Burkhalter A. Buzàki G. Cauli B. Defelipe J. Faién A. Feldmeyer D. Fishell G. Fregnac Y. Freund T. F. Gardner E. P. Goldberg J. H. Helmstaedter M. Hestrin S. Karube F. Kisvàrday Z. F. Lambolez B. Lewis D. A. Marin O. Markram H. Munoz A. Packer A. Petersen C. C. Rockland K. S. Rossier J. Rudy B. Somogy P. Staiger J. F. Tamas G. Thomson A. M. Toledo-Rodriguez M. Wang Y. West D. C. Yuste R. (2008). Petilla terminology: nomenclature of features of GABAergic interneurons of the cerebral cortex. Nat. Rev. Neurosci. 9, 557–568 10.1038/nrn2402 18568015 PMC2868386

[B5] Baraban S. C. Tallent M. K. (2004). Interneuron diversity series: interneuronal neuropeptides– endogenous regulators of neuronal excitability. Trends Neurosci. 27, 135–142 10.1016/j.tins.2004.01.008 15036878

[B6] Barnes N. M. Sharp T. (1999). A review of central 5-HT receptors and their function. Neuropharmacology 38, 1083–1152 10.1016/S0028-3908(99)00010-6 10462127

[B7] Batista C. M. De Paula K. C. Cavalcante L. A. Mendez-Otero R. (2001). Subcellular localization of neuronal nitric oxide synthase in the superficial gray layer of the rat superior colliculus. Neurosci. Res. 41, 67–70 10.1016/S0168-0102(01)00268-1 11535295

[B8] Beaulieu C. (1993). Numerical data on neocortical neurons in adult rat, with special reference to the GABA population. Brain Res. 609, 284–292 10.1016/0006-8993(93)90884-P 8508310

[B9] Bredt D. S. Glatt C. E. Hwang P. M. Fotuhi M. Dawson T. M. Snyder S. H. (1991). Nitric oxide synthase protein and mRNA are discretely localized in neuronal populations of the mammalian CNS together with NADPH diaphorase. Neuron 7, 615–624 10.1016/0896-6273(91)90374-9 1718335

[B10] Brown L. A. Key B. J. Lovick T. A. (2000). Fluorescent imaging of nitric oxide production in neuronal varicosities associated with intraparenchymal arterioles in rat hippocampal slices. Neurosci. Lett. 294, 9–12 11044574 10.1016/s0304-3940(00)01521-4

[B11] Cauli B. Audinat E. Lambolez B. Angulo M. C. Ropert N. Tsuzuki K. Hestrin S. Rossier J. (1997). Molecular and physiological diversity of cortical nonpyramidal cells. J. Neurosci. 17, 3894–3906 9133407 10.1523/JNEUROSCI.17-10-03894.1997 PMC6573690

[B12] Cauli B. Hamel E. (2010). Revisiting the role of neurons in neurovascular coupling. Front. Neuroenergetics 2:9 10.3389/fnene.2010.00009 20616884 PMC2899521

[B13] Cauli B. Tong X. K. Rancillac A. Serluca N. Lambolez B. Rossier J. Hamel E. (2004). Cortical GABA interneurons in neurovascular coupling: relays for subcortical vasoactive pathways. J. Neurosci. 24, 8940–8949 10.1523/JNEUROSCI.3065-04.2004 15483113 PMC6730057

[B14] Chameau P. van Hooft J. A. (2006). Serotonin 5-HT(3) receptors in the central nervous system. Cell Tissue Res. 326, 573–581 10.1007/s00441-006-0255-8 16826372

[B15] Chédotal A. Cozzari C. Faure M. P. Hartman B. K. Hamel E. (1994a). Distinct choline acetyltransferase (ChAT) and vasoactive intestinal polypeptide (VIP) bipolar neurons project to local blood vessels in the rat cerebral cortex. Brain Res. 646, 181–193 8069662 10.1016/0006-8993(94)90076-0

[B16] Chédotal A. Umbriaco D. Descarries L. Hartman B. K. Hamel E. (1994b). Light and electron microscopic immunocytochemical analysis of the neurovascular relationships of choline acetyltransferase and vasoactive intestinal polypeptide nerve terminals in the rat cerebral cortex. J. Comp. Neurol. 343, 57–71 10.1002/cne.903430105 8027437

[B17] Cho K. H. Jang J. H. Jang H. J. Kim M. J. Yoon S. H. Fukuda T. Tennigkeit F. Singer W. Rhie D. J. (2010). Subtype-specific dendritic Ca(2+) dynamics of inhibitory interneurons in the rat visual cortex. J. Neurophysiol. 104, 840–853 10.1152/jn.00146.2010 20554844

[B18] Cohen Z. Bonvento G. Lacombe P. Hamel E. (1996). Serotonin in the regulation of brain microcirculation. Prog. Neurobiol. 50, 335–362 10.1016/S0301-0082(96)00033-0 9004349

[B19] Dacey R. G. Jr. Bassett J. E. Takayasu M. (1988). Vasomotor responses of rat intracerebral arterioles to vasoactive intestinal peptide, substance P, neuropeptide Y, and bradykinin. J. Cereb. Blood Flow Metab. 8, 254–261 10.1038/jcbfm.1988.56 2449445

[B20] DeFelipe J. Hendry S. H. Hashikawa T. Jones E. G. (1991). Synaptic relationships of serotonin-immunoreactive terminal baskets on GABA neurons in the cat auditory cortex. Cereb. Cortex 1, 117–133 10.1093/cercor/1.2.117 1822729

[B21] Eckenstein F. Baughman R. W. (1984). Two types of cholinergic innervation in cortex, one co-localized with vasoactive intestinal polypeptide. Nature 309, 153–155 6717593 10.1038/309153a0

[B22] Estrada C. DeFelipe J. (1998). Nitric oxide-producing neurons in the neocortex: morphological and functional relationship with intraparenchymal microvasculature. Cereb. Cortex 8, 193–203 9617914 10.1093/cercor/8.3.193

[B23] Fahrenkrug J. Hannibal J. Tams J. Georg B. (2000). Immunohistochemical localization of the VIP1 receptor (VPAC1R) in rat cerebral blood vessels: relation to PACAP and VIP containing nerves. J. Cereb. Blood Flow Metab. 20, 1205–1214 10.1097/00004647-200008000-00006 10950381

[B24] Fanselow E. E. Connors B. W. (2010). The roles of somatostatin-expressing (GIN) and fast-spiking inhibitory interneurons in UP-DOWN states of mouse neocortex. J. Neurophysiol. 104, 596–606 10.1152/jn.00206.2010 20538767 PMC2934925

[B25] Ferezou I. Cauli B. Hill E. L. Rossier J. Hamel E. Lambolez B. (2002). 5-HT3 receptors mediate serotonergic fast synaptic excitation of neocortical vasoactive intestinal peptide/cholecystokinin interneurons. J. Neurosci. 22, 7389–7397 12196560 10.1523/JNEUROSCI.22-17-07389.2002 PMC6757992

[B25a] Foehring R. C. Van Brederode J. F. Kinney G. A. Spain W. J. (2002). Serotonergic modulation of supragranular neurons in rat sensorimotor cortex. J. Neurosci. 22, 8238–8250 12223578 10.1523/JNEUROSCI.22-18-08238.2002 PMC6758114

[B26] Gabbott P. L. Dickie B. G. Vaid R. R. Headlam A. J. Bacon S. J. (1997). Local-circuit neurones in the medial prefrontal cortex (areas 25, 32 and 24b) in the rat: morphology and quantitative distribution. J. Comp. Neurol. 377, 465–499 10.1002/(SICI)1096-9861(19970127) 9007187

[B27] Garthwaite J. (2008). Concepts of neural nitric oxide-mediated transmission. Eur. J. Neurosci. 27, 2783–2802 10.1111/j.1460-9568.2008.06285.x 18588525 PMC2610389

[B28] Gentet L. J. Avermann M. Matyas F. Staiger J. F. Petersen C. C. (2010). Membrane potential dynamics of GABAergic neurons in the barrel cortex of behaving mice. Neuron 65, 422–435 10.1016/j.neuron.2010.01.006 20159454

[B29] Gholipour T. Ghasemi M. Riazi K. Ghaffarpour M. Dehpour A. R. (2010). Seizure susceptibility alteration through 5-HT(3) receptor: modulation by nitric oxide. Seizure 19, 17–22 10.1016/j.seizure.2009.10.006 19942458

[B30] Heintz N. (2001). BAC to the future: the use of bac transgenic mice for neuroscience research. Nat. Rev. Neurosci. 2, 861–870 10.1038/35104049 11733793

[B31] Heintz N. (2004). Gene expression nervous system atlas (GENSAT). Nat. Neurosci. 7, 483 10.1038/nn0504-483 15114362

[B32] Hendricks T. Francis N. Fyodorov D. Deneris E. S. (1999). The ETS domain factor Pet-1 is an early and precise marker of central serotonin neurons and interacts with a conserved element in serotonergic genes. J. Neurosci. 19, 10348–10356 10575032 10.1523/JNEUROSCI.19-23-10348.1999 PMC6782418

[B33] Hendricks T. J. Fyodorov D. V. Wegman L. J. Lelutiu N. B. Pehek E. A. Yamamoto B. Silver J. Weeber E. J. Sweatt J. D. Deneris E. S. (2003). Pet-1 ETS gene plays a critical role in 5-HT neuron development and is required for normal anxiety-like and aggressive behavior. Neuron 37, 233–247 10.1016/S0896-6273(02)01167-4 12546819

[B34] Hoyer D. Clarke D. E. Fozard J. R. Hartig P. R. Martin G. R. Mylecharane E. J. Saxena P. R. Humphrey P. P. (1994). International union of pharmacology classification of receptors for 5-hydroxytryptamine (Serotonin). Pharmacol. Rev. 46, 157–203 7938165

[B35] Jackson M. B. Yakel J. L. (1995). The 5-HT3 receptor channel. Annu. Rev. Physiol. 57, 447–468 10.1146/annurev.ph.57.030195.002311 7539990

[B36] Jakab R. L. Goldman-Rakic P. S. (2000). Segregation of serotonin 5-HT2A and 5-HT3 receptors in inhibitory circuits of the primate cerebral cortex. J. Comp. Neurol. 417, 337–348 10683608 10.1002/(sici)1096-9861(20000214)417:3<337::aid-cne7>3.0.co;2-o

[B37] Karagiannis A. Gallopin T. David C. Battaglia D. Geoffroy H. Rossier J. Hillman E. M. Staiger J. F. Cauli B. (2009). Classification of NPY-expressing neocortical interneurons. J. Neurosci. 29, 3642–3659 10.1523/JNEUROSCI.0058-09.2009 19295167 PMC2750888

[B38] Kitaura H. Uozumi N. Tohmi M. Yamazaki M. Sakimura K. Kudoh M. Shimizu T. Shibuki K. (2007). Roles of nitric oxide as a vasodilator in neurovascular coupling of mouse somatosensory cortex. Neurosci. Res. 59, 160–171 10.1016/j.neures.2007.06.1469 17655958

[B39] Kits K. S. Mansvelder H. D. (2000). Regulation of exocytosis in neuroendocrine cells: spatial organization of channels and vesicles, stimulus-secretion coupling, calcium buffers and modulation. Brain Res. Brain Res. Rev. 33, 78–94 10.1016/S0165-0173(00)00023-0 10967354

[B40] Kiyasova V. Fernandez S. P. Laine J. Stankovski L. Muzerelle A. Doly S. Gaspar P. (2011). A genetically defined morphologically and functionally unique subset of 5-HT neurons in the mouse raphe nuclei. J. Neurosci. 31, 2756–2768 10.1523/JNEUROSCI.4080-10.2011 21414898 PMC6623784

[B41] Krnjevic K. Phillis J. W. (1963). Iontophoretic studies of neurones in the mammalian cerebral cortex. J. Physiol. 165, 274–304 14035891 10.1113/jphysiol.1963.sp007057 PMC1359271

[B42] Kubota Y. Shigematsu N. Karube F. Sekigawa A. Kato S. Yamaguchi N. Hirai Y. Morishima M. Kawaguchi Y. (2011). Selective coexpression of multiple chemical markers defines discrete populations of neocortical GABAergic neurons. Cereb. Cortex 21, 1803–1817 10.1093/cercor/bhq252 21220766

[B43] Lee S. Hjerling-Leffler J. Zagha E. Fishell G. Rudy B. (2010). The largest group of superficial neocortical GABAergic interneurons expresses ionotropic serotonin receptors. J. Neurosci. 30, 16796–16808 10.1523/JNEUROSCI.1869-10.2010 21159951 PMC3025500

[B44] Liu C. Maejima T. Wyler S. C. Casadesus G. Herlitze S. Deneris E. S. (2010). Pet-1 is required across different stages of life to regulate serotonergic function. Nat. Neurosci. 13, 1190–1198 10.1038/nn.2623 20818386 PMC2947586

[B45] Liu X. Li C. Falck J. R. Roman R. J. Harder D. R. Koehler R. C. (2008). Interaction of nitric oxide, 20-HETE, and EETs during functional hyperemia in whisker barrel cortex. Am. J. Physiol. Heart Circ. Physiol. 295, H619–H631 10.1152/ajpheart.01211.2007 18502903 PMC2519225

[B46] Lovick T. A. Brown L. A. Key B. J. (1999). Neurovascular relationships in hippocampal slices: physiological and anatomical studies of mechanisms underlying flow-metabolism coupling in intraparenchymal microvessels. Neuroscience 92, 47–60 10.1016/S0306-4522(98)00737-4 10392829

[B47] Ludwig M. Pittman Q. J. (2003). Talking back: dendritic neurotransmitter release. Trends Neurosci. 26, 255–261 10.1016/S0166-2236(03)00072-9 12744842

[B48] Ludwig M. Sabatier N. Bull P. M. Landgraf R. Dayanithi G. Leng G. (2002). Intracellular calcium stores regulate activity-dependent neuropeptide release from dendrites. Nature 418, 85–89 10.1038/nature00822 12097911

[B49] Markram H. Toledo-Rodriguez M. Wang Y. Gupta A. Silberberg G. Wu C. (2004). Interneurons of the neocortical inhibitory system. Nat. Rev. Neurosci. 5, 793–807 10.1038/nrn1519 15378039

[B50] McCulloch J. Edvinsson L. (1980). Cerebral circulatory and metabolic effects of vasoactive intestinal polypeptide. Am. J. Physiol. 238, H449–H456 7377315 10.1152/ajpheart.1980.238.4.H449

[B51] Mendez P. Bacci A. (2011). Assortment of GABAergic plasticity in the cortical interneuron melting pot. Neural Plast. 2011, 976856 10.1155/2011/976856 21785736 PMC3139185

[B52] Morales M. Battenberg E. De L. L. Sanna P. P. Bloom F. E. (1996). Cellular and subcellular immunolocalization of the type 3 serotonin receptor in the rat central nervous system. Brain Res. Mol. Brain Res. 36, 251–260 10.1016/0169-328X(96)88406-3 8965645

[B53] Morales M. Bloom F. E. (1997). The 5-HT3 receptor is present in different subpopulations of GABAergic neurons in the rat telencephalon. J. Neurosci. 17, 3157–3167 9096150 10.1523/JNEUROSCI.17-09-03157.1997 PMC6573651

[B54] Moreau A. W. Amar M. Le R. N. Morel N. Fossier P. (2010). Serotoninergic fine-tuning of the excitation-inhibition balance in rat visual cortical networks. Cereb. Cortex 20, 456–467 10.1093/cercor/bhp114 19520765

[B55] Nayak S. V. Ronde P. Spier A. D. Lummis S. C. Nichols R. A. (1999). Calcium changes induced by presynaptic 5-hydroxytryptamine-3 serotonin receptors on isolated terminals from various regions of the rat brain. Neuroscience 91, 107–117 10.1016/S0306-4522(98)00520-X 10336063

[B56] Nishimura N. Schaffer C. B. Friedman B. Lyden P. D. Kleinfeld D. (2007). Penetrating arterioles are a bottleneck in the perfusion of neocortex. Proc. Natl. Acad. Sci. U.S.A. 104, 365–370 10.1073/pnas.0609551104 17190804 PMC1765467

[B57] Papadopoulos G. C. Parnavelas J. G. Buijs R. M. (1987). Light and electron microscopic immunocytochemical analysis of the serotonin innervation of the rat visual cortex. J. Neurocytol. 16, 883–892 3450795 10.1007/BF01611992

[B58] Paspalas C. D. Papadopoulos G. C. (2001). Serotoninergic afferents preferentially innervate distinct subclasses of peptidergic interneurons in the rat visual cortex. Brain Res. 891, 158–167 10.1016/S0006-8993(00)03193-0 11164819

[B59] Perrenoud Q. Geoffroy H. Gauthier B. Rancillac A. Kessaris N. Rossier J. Vitalis T. Gallopin T. (2012). Characterization of Type I and Type II nNOS expressing neurons in the barrel cortex of mouse. Front. Neuronal Circuits 6:36 10.3389/fncir.2012.00036 22754499 PMC3386492

[B60] Petzold G. C. Murthy V. N. (2011). Role of astrocytes in neurovascular coupling. Neuron 71, 782–797 10.1016/j.neuron.2011.08.009 21903073

[B61] Porter J. T. Cauli B. Tsuzuki K. Lambolez B. Rossier J. Audinat E. (1999). Selective excitation of subtypes of neocortical interneurons by nicotinic receptors. J. Neurosci. 19, 5228–5235 10377334 10.1523/JNEUROSCI.19-13-05228.1999 PMC6782331

[B62] Puig M. V. Artigas F. Celada P. (2005). Modulation of the activity of pyramidal neurons in rat prefrontal cortex by raphe stimulation *in vivo*: involvement of serotonin and GABA. Cereb. Cortex 15, 1–14 10.1093/cercor/bhh104 15238448

[B63] Pytliak M. Vargova V. Mechirova V. Felsoci M. (2011). Serotonin receptors—from molecular biology to clinical applications. Physiol. Res. 60, 15–25 20945968 10.33549/physiolres.931903

[B63a] Rancillac A. Goeffroy H. Rossier J. (2012). Impaired neurovascular coupling in the APPxPS1 mouse model of Alzheimer's disease. Curr. Alzheimer Res. [Epub ahead of print]. 22799606 10.2174/156720512804142859

[B64] Rancillac A. Lainé J. Perrenoud Q. Geoffroy H. Ferezou I. Vitalis T. Rossier J. (2010). Degenerative abnormalities in transgenic neocortical neuropeptide Y interneurons expressing tau-green fluorescent protein. J. Neurosci. Res. 88, 487–99 10.1002/jnr.22234 19830842

[B65] Rancillac A. Rossier J. Guille M. Tong X. K. Geoffroy H. Amatore C. Arbault S. Hamel E. Cauli B. (2006). Glutamatergic control of microvascular tone by distinct GABA neurons in the cerebellum. J. Neurosci. 26, 6997–7006 10.1523/JNEUROSCI.5515-05.2006 16807329 PMC6673912

[B66] Rapport M. M. Green A. A. Page I. H. (1948). Serum vasoconstrictor, serotonin; isolation and characterization. J. Biol. Chem. 176, 1243–1251 18100415

[B67] Reader T. A. (1978). The effects of dopamine, noradrenaline and serotonin in the visual cortex of the cat. Experientia 34, 1586–1588 729721 10.1007/BF02034690

[B68] Reinhard J. F. Jr. Liebmann J. E. Schlosberg A. J. Moskowitz M. A. (1979). Serotonin neurons project to small blood vessels in the brain. Science 206, 85–87 10.1126/science.482930 482930

[B69] Riad M. Tong X. K. El M. S. Hamon M. Hamel E. Descarries L. (1998). Endothelial expression of the 5-hydroxytryptamine1B antimigraine drug receptor in rat and human brain microvessels. Neuroscience 86, 1031–1035 10.1016/S0306-4522(98)00196-1 9697110

[B70] Roerig B. Nelson D. A. Katz L. C. (1997). Fast synaptic signaling by nicotinic acetylcholine and serotonin 5-HT3 receptors in developing visual cortex. J. Neurosci. 17, 8353–8362 9334409 10.1523/JNEUROSCI.17-21-08353.1997 PMC6573745

[B71] Rossier J. (2009). Wiring and plumbing in the brain. Front. Hum. Neurosci. 3:2 10.3389/neuro.09.002.2009 19287480 PMC2649200

[B72] Rudy B. Fishell G. Lee S. Hjerling-Leffler J. (2011). Three groups of interneurons account for nearly 100% of neocortical GABAergic neurons. Dev. Neurobiol. 71, 45–61 10.1002/dneu.20853 21154909 PMC3556905

[B73] Sagher O. Zhang X. Q. Szeto W. Thai Q. A. Jin Y. Kassell N. F. Lee K. S. (1993). Live computerized videomicroscopy of cerebral microvessels in brain slices. J. Cereb. Blood Flow Metab. 13, 676–682 10.1038/jcbfm.1993.86 8314920

[B74] Simpson K. L. Waterhouse B. D. Lin R. C. (2003). Differential expression of nitric oxide in serotonergic projection neurons: neurochemical identification of dorsal raphe inputs to rodent trigeminal somatosensory targets. J. Comp. Neurol. 466, 495–512 10.1002/cne.10912 14566945

[B75] Smiley J. F. Goldman-Rakic P. S. (1996). Serotonergic axons in monkey prefrontal cerebral cortex synapse predominantly on interneurons as demonstrated by serial section electron microscopy. J. Comp. Neurol. 367, 431–443 10.1002/(SICI)1096-9861(19960408)367:3<431::AID-CNE8>3.0.CO;2-6 8698902

[B76] Steinbusch H. W. (1981). Distribution of serotonin-immunoreactivity in the central nervous system of the rat-cell bodies and terminals. Neuroscience 6, 557–618 7017455 10.1016/0306-4522(81)90146-9

[B77] Sudweeks S. N. Hooft J. A. Yakel J. L. (2002). Serotonin 5-HT(3) receptors in rat CA1 hippocampal interneurons: functional and molecular characterization. J. Physiol. 544, 715–726 10.1113/jphysiol.2002.029736 12411518 PMC2290631

[B78] Takeuchi Y. Sano Y. (1984). Serotonin nerve fibers in the primary visual cortex of the monkey. Quantitative and immunoelectronmicroscopical analysis. Anat. Embryol. (Berl.) 169, 1–8 6721216 10.1007/BF00300581

[B79] Tong X. K. Hamel E. (2000). Basal forebrain nitric oxide synthase (NOS)-containing neurons project to microvessels and NOS neurons in the rat neocortex: cellular basis for cortical blood flow regulation. Eur. J. Neurosci. 12, 2769–2780 10.1046/j.1460-9568.2000.00158.x 10971619

[B80] Tork I. (1990). Anatomy of the serotonergic system. Ann. N.Y. Acad. Sci. 600, 9–34 10.1111/j.1749-6632.1990.tb16870.x 2252340

[B81] Underwood M. D. Bakalian M. J. Arango V. Smith R. W. Mann J. J. (1992). Regulation of cortical blood flow by the dorsal raphe nucleus: topographic organization of cerebrovascular regulatory regions. J. Cereb. Blood Flow Metab. 12, 664–673 10.1038/jcbfm.1992.91 1618944

[B82] van Hooft J. A. Spier A. D. Yakel J. L. Lummis S. C. Vijverberg H. P. (1998). Promiscuous coassembly of serotonin 5-HT3 and nicotinic alpha4 receptor subunits into Ca(2+)-permeable ion channels. Proc. Natl. Acad. Sci. U.S.A. 95, 11456–11461 10.1073/pnas.95.19.11456 9736758 PMC21664

[B83] Vitalis T. Rossier J. (2011). New insights into cortical interneurons development and classification: contribution of developmental studies. Dev. Neurobiol. 71, 34–44 10.1002/dneu.20810 21154908

[B84] Vucurovic K. Gallopin T. Ferezou I. Rancillac A. Chameau P. van Hooft J. A. Geoffroy H. Monyer H. Rossier J. Vitalis T. (2010). Serotonin 3A receptor subtype as an early and protracted marker of cortical interneuron subpopulations. Cereb. Cortex 20, 2333–2347 10.1093/cercor/bhp310 20083553 PMC2936799

[B85] Walstab J. Rappold G. Niesler B. (2010). 5-HT(3) receptors: role in disease and target of drugs. Pharmacol. Ther. 128, 146–169 10.1016/j.pharmthera.2010.07.001 20621123

[B86] Waterhouse B. D. Azizi S. A. Burne R. A. Woodward D. J. (1990). Modulation of rat cortical area 17 neuronal responses to moving visual stimuli during norepinephrine and serotonin microiontophoresis. Brain Res. 514, 276–292 2357542 10.1016/0006-8993(90)91422-d

[B87] Whittington M. A. Traub R. D. (2003). Interneuron diversity series: inhibitory interneurons and network oscillations *in vitro*. Trends Neurosci. 26, 676–682 10.1016/j.tins.2003.09.016 14624852

[B88] Yaksh T. L. Wang J. Y. Go V. L. (1987). Cortical vasodilatation produced by vasoactive intestinal polypeptide (VIP) and by physiological stimuli in the cat. J. Cereb. Blood Flow Metab. 7, 315–326 10.1038/jcbfm.1987.69 3108270

[B89] Yan X. X. Jen L. S. Garey L. J. (1996). NADPH-diaphorase-positive neurons in primate cerebral cortex colocalize with GABA and calcium-binding proteins. Cereb. Cortex 6, 524–529 10.1093/cercor/6.3.524 8670678

[B90] Yang G. Chen G. Ebner T. J. Iadecola C. (1999). Nitric oxide is the predominant mediator of cerebellar hyperemia during somatosensory activation in rats. Am. J. Physiol. 277, R1760–R1770 10600924 10.1152/ajpregu.1999.277.6.R1760

[B91] Yang G. Huard J. M. Beitz A. J. Ross M. E. Iadecola C. (2000). Stellate neurons mediate functional hyperemia in the cerebellar molecular layer. J. Neurosci. 20, 6968–6973 10995841 10.1523/JNEUROSCI.20-18-06968.2000 PMC6772810

[B92] Zhou F. M. Hablitz J. J. (1999). Activation of serotonin receptors modulates synaptic transmission in rat cerebral cortex. J. Neurophysiol. 82, 2989–2999 10601434 10.1152/jn.1999.82.6.2989

[B93] Zupanc G. K. (1996). Peptidergic transmission: from morphological correlates to functional implications. Micron 27, 35–91 10.1016/0968-4328(95)00028-3 8756315

